# Jaundice in patients with COVID‐19

**DOI:** 10.1002/jgh3.12645

**Published:** 2021-08-24

**Authors:** Joshua M Bender, Howard J Worman

**Affiliations:** ^1^ Department of Medicine, Vagelos College of Physicians and Surgeons Columbia University New York New York USA; ^2^ Department of Pathology and Cell Biology, Vagelos College of Physicians and Surgeons Columbia University New York New York USA

**Keywords:** bilirubin, COVID‐19, jaundice, liver, liver injury, severe acute respiratory syndrome coronavirus 2

## Abstract

**Background and Aim:**

While many studies have reported on liver injury in patients with coronavirus disease 2019 (COVID‐19), none have specifically addressed the significance of hepatic jaundice. We aimed to determine the clinical consequences and etiologies of jaundice in patients with COVID‐19.

**Methods:**

We retrospectively analyzed clinical features, laboratory abnormalities, and rates of survival and intensive care unit admission in 551 patients with COVID‐19, hospitalized between 1 March 2020, and 31 May 2020 at a tertiary care academic medical center. Hepatic jaundice was defined as a serum total bilirubin concentration >2.5 mg/dL and a direct bilirubin concentration >0.3 mg/dL that was >25% of the total. Liver injury was characterized as cholestatic, mixed, or hepatocellular at the time of peak serum total bilirubin concentration by calculating the R factor.

**Results:**

Hepatic jaundice was present in 49 (8.9%) patients and associated with a mortality rate of 40.8% and intensive care unit admission rate of 69.4%, both significantly higher than for patients without jaundice. Jaundiced patients had an increased frequency of fever, leukopenia, leukocytosis, thrombocytopenia, hypotension, hypoxemia, elevated serum creatinine concentration, elevated serum procalcitonin concentration, and sepsis. Nine jaundiced patients had isolated hyperbilirubinemia. Of the 40 patients with abnormally elevated serum alanine aminotransferase or alkaline phosphatase activities, 62.5% had a cholestatic, 20.0% mixed, and 17.5% hepatocellular pattern of liver injury.

**Conclusion:**

Hepatic jaundice in patients with COVID‐19 is associated with high mortality. The main etiologies of liver dysfunction leading to jaundice appear to be sepsis, severe systemic inflammation, and hypoxic/ischemic hepatitis.

## Graphical abstract

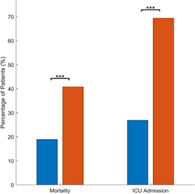

Jaundice is a hallmark of liver dysfunction. We show that hepatic jaundice in patients with COVID‐19 is associated with high mortality, similar to that observed with invasive mechanical ventilation. The main etiologies of liver dysfunction leading to jaundice appear to be sepsis, severe systemic inflammation, and hypoxic/ischemic hepatitis.

## Introduction

Jaundice is a hallmark of liver dysfunction. Hepatocytes take up unconjugated bilirubin, a product of heme metabolism, conjugate it to bilirubin diglucuronide, and excrete it into the bile.^1^, ^2^, ^3^ Conjugated bilirubin is found in serum only in the presence of hepatic dysfunction (or in the ultrarare Rotor and Dubin–Johnson syndromes). Serum direct bilirubin concentration measured in the clinical laboratory correlates with the conjugated fraction. However, because of the detection methods utilized, unconjugated bilirubin could account for up to 25% of the serum direct bilirubin concentration. Although there is variability between observers and no single quantitative definition, jaundice can generally be detected at a serum total bilirubin concentration (TBIL) from 2.5 to 3.0 mg/dL.^3^, ^4^, ^5^

Severe acute respiratory syndrome coronavirus 2 (SARS‐CoV‐2) is the virus that causes coronavirus disease 2019 (COVID‐19), the respiratory illness responsible for a global pandemic and millions of deaths worldwide. Many published studies have reported on liver injury and liver‐related blood test abnormalities in patients with COVID‐19.^6^ However, outcomes associated with and causes of jaundice have not been specifically evaluated. We previously found that TBIL or serum direct bilirubin concentration above the laboratory upper limit of normal (ULN) was associated with increased mortality in patients with COVID‐19.^7^ We therefore examined the clinical features and outcomes of hospitalized patients with COVID‐19 who had serum direct bilirubin concentrations high enough to cause jaundice and identified common pathologies that led to liver dysfunction.

## Methods

### Inclusion criteria and data collection

The Columbia University Institutional Review Board approved the protocol with a waiver of informed consent. Participants included in the study were admitted to NewYork‐Presbyterian Hospital/Columbia University Irving Medical Center (CUIMC) between 1 March 2020, and 31 May 2020, with an encounter diagnosis of COVID‐19 (International Classification of Diseases, Tenth Revision code U07.1). This code is only used for a confirmed diagnosis of COVID‐19 as documented by the provider. We used this eligibility criterion to include only patients who suffered from the disease caused by SARS‐CoV‐2 and exclude those who may have tested positive while admitted for other reasons. All subjects had a positive RT‐PCR nasal swab for SARS‐CoV‐2 RNA.

Patient demographics, laboratory values, vital signs, clinical outcomes, and medical histories were obtained by query of the Epic Systems electronic health record, and outcomes were assessed at the time of data collection on 21 July 2020. Race and ethnicity were self‐reported in prespecified categories. Laboratory test abnormalities, per CUIMC laboratory reference ranges, were defined as follows: alanine aminotransferase (ALT) >50 U/L, alkaline phosphatase (ALP) >129 U/L, direct bilirubin >0.3 mg/dL, platelet count <156 000/μL, leukocyte cell count <3120 or >8440/μL, creatinine >1.3 mg/dL, C‐reactive protein >10.0 mg/L, erythrocyte sedimentation rate >15 mm/h, and procalcitonin >0.25 ng/mL. Hypotension was defined as a mean arterial pressure <60 mmHg and hypoxia as arterial oxygen saturation <90%.

### Criteria for hepatic jaundice, SIRS, and sepsis

Criteria for hepatic jaundice were a TBIL >2.5 mg/dL and a serum direct bilirubin concentration >0.3 mg/dL that was also >25% of TBIL. These criteria excluded patients with indirect hyperbilirubinemia from causes other than acquired liver dysfunction, such as hemolysis or Gilbert syndrome. While there is no precise value for TBIL causing detectable jaundice, we used 2.5 mg/dL, which is a frequently cited lower limit.^4^, ^5^ Patients were determined to have systemic inflammatory response syndrome (SIRS) if, at any point during their hospitalization, they satisfied two or more of the following criteria: (i) body temperature over 38°C or under 36°C, (ii) heart rate >90 beats/min, (iii) respiratory rate >20 breaths/min or partial pressure of CO_2_ <32 mmHg, (iv) leukocyte count >12 000/μL or <4000/μL or over 10% immature forms or bands. Sepsis was defined as SIRS with a suspected source of infection.^8^ In this study, positive blood cultures were considered evidence of infection. Blood cultures that isolated only *S. epidermidis* were excluded due to the high frequency of contamination.^9^

### Characterization of liver injury

Liver injury was characterized as cholestatic, mixed, or hepatocellular at the time of peak TBIL by calculating the R factor. The R factor is equal to serum ALT/ULN divided by serum ALP/ULN. R ≥ 5 is considered hepatocellular liver injury, R ≤ 2 cholestatic, and 2 < R < 5 as a mixed type of liver injury.^10^, ^11^

### Statistical analyses

All analyses were performed using MATLAB R2020a (version 9.8.0.1396136; The MathWorks, Inc., Natick, MA, USA). Categorical variables were compared using Chi‐square analysis or Tukey's honest significant difference test, as appropriate. *P* values ≤0.05 were considered statistically significant.

## Results

### Demographics, outcomes, and clinical features of patients with COVID‐19 and hepatic jaundice

Of 551 patients fitting the inclusion criteria for having COVID‐19, 49 (8.9%) also met the criteria for having hepatic jaundice. There were a few demographic differences between the patients with hepatic jaundice and those without (Table 1). The proportion of jaundiced male patients was significantly higher than the proportion of non‐jaundiced ones (75.5 *vs* 55.6%; *P* = 0.007). Additionally, there was a significantly higher proportion of Hispanic/Latino (65.3 *vs* 50.1%; *P* = 0.04) and obese patients (42.9 *vs* 25.7%; *P* = 0.01) with jaundice.

**Table 1:** Demographics of jaundiced and non‐jaundiced patients with COVID‐19

	Non‐jaundiced	Jaundiced
No. of patients in cohort	502	49
Age		
<25	27 (5.4%)	4 (8.2%)
25–49	77 (15.3%)	9 (18.4%)
50–64	133 (26.5%)	17 (34.7%)
65–79	177 (35.3%)	17 (34.7%)
>80	88 (17.5%)	2 (4.1%)
Sex		
Female	223 (44.4%)	12 (24.5%)**
Male	279 (55.6%)	37 (75.5%)**
Race		
Asian	9 (1.8%)	0 (0.0%)
African American	92 (18.3%)	9 (18.4%)
White	123 (24.5%)	9 (18.4%)
Other/multiracial	178 (35.5%)	19 (38.8%)
Declined	100 (19.9%)	12 (24.5%)
Ethnicity (Latino yes/no)		
Hispanic or Latino	252 (50.1%)	32 (65.3%)*
Not Hispanic or Latino	158 (31.4%)	12 (24.5%)
Declined	92 (18.3%)	5 (10.2%)
Body mass index (kg/m^2^)		
Underweight (<18.5)	19 (3.8%)	1 (2.0%)
Normal (18.5–24.9)	120 (23.9%)	7 (14.3%)
Overweight (25–29.9)	140 (27.9%)	13 (26.5%)
Obese (30–39.9)	129 (25.7%)	21 (42.9%)*
Extremely Obese (>40)	34 (6.8%)	6 (12.2%)
Unknown	60 (12.0%)	1 (2.0%)

* *P* < 0.05,

** *P* < 0.01.

**Figure 1:**
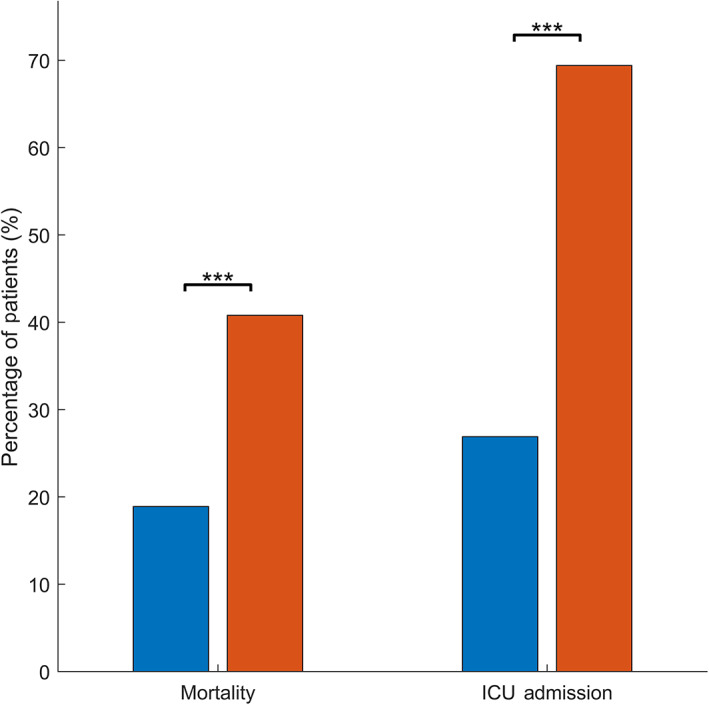
Mortality and intensive care unit (ICU) admission rates in non‐jaundiced and jaundiced patients hospitalized with COVID‐19. ***P < 0.001. (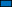), Non‐jaundiced; (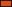), jaundiced.

Hepatic jaundice in hospitalized patients with COVID‐19 was associated with significantly worse clinical outcomes (Fig. 1). The mortality rate among jaundiced patients was significantly higher than that in patients without evidence of jaundice (40.8 *vs* 18.9%, *P* < 0.001). Additionally, patients with jaundice were admitted to the intensive care unit (ICU) more frequently than those without jaundice (69.4 *vs* 26.9%, *P* < 0.001).

In addition to adverse clinical outcomes, jaundice in patients with COVID‐19 was associated with various historical, clinical, and laboratory abnormalities (Table 2). Patients with a history of liver disease were more likely to be jaundiced than those without a prior history of liver disease. Jaundiced patients had an increased frequency of fever, leukopenia, leukocytosis, thrombocytopenia, hypotension, and hypoxemia. They also had an increased frequency of other laboratory abnormalities including elevated serum creatinine concentration, elevated serum procalcitonin concentration, positive blood cultures, and sepsis. SIRS, however, was present in approximately 90% of all patients, whether or not they were jaundiced.

**Table 2:** Clinical and laboratory parameters in non‐jaundiced and jaundiced patients with COVID‐19

	Non‐jaundiced	Jaundiced	*P* value
Number of patients	502	49	
History of liver disease	24 (4.8%)	8 (16.3%)	<0.001
Fever	315 (62.8%)	39 (79.6%)	0.019
Leukopenia	46 (9.2%)	9 (18.4%)	0.040
Leukocytosis	380 (75.7%)	47 (96.0%)	0.001
Thrombocytopenia	217 (43.2%)	36 (73.5%)	<0.001
Hypotension	166 (33.1%)	33 (67.4%)	<0.001
Hypoxia	346 (68.9%)	43 (87.8%)	0.006
Elevated creatinine	253 (50.4%)	34 (69.4%)	0.011
Elevated C‐reactive protein	446 (88.8%)	47 (95.9%)	0.124
Prolonged ESR	436 (86.9%)	44 (89.8%)	0.557
Elevated procalcitonin	309 (61.6%)	42 (85.7%)	0.001
Positive blood culture	40 (8.0%)	12 (24.5%)	<0.001
SIRS	449 (89.4%)	45 (91.8%)	0.599
Sepsis	39 (7.8%)	12 (24.5%)	<0.001

ESR, erythrocyte sedimentation rate.

### Characterization of liver injury in patients with COVID‐19 and hepatic jaundice

**Figure 2:**
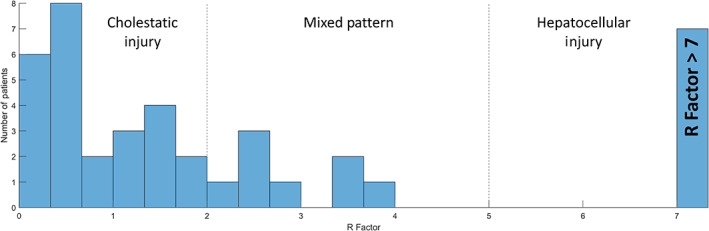
R factor of 40 jaundiced patients hospitalized with COVID‐19 with an elevated alanine aminotransferase and/or alkaline phosphatase at the time of peak serum total bilirubin concentration. Seven patients had an R factor > 7, ranging from 9.19 to 127; these are represented by the bar labeled “R Factor > 7”.

The type of liver injury in patients with jaundice was characterized as hepatocellular, mixed, or cholestatic by calculating the R factor at the time that TBIL peaked. Nine jaundiced patients (18.4%) had isolated hyperbilirubinemia with normal ALT and ALP; therefore, we calculated the R factor for the 40 with an abnormal ALT or ALP at the time of peak TBIL. Of these, 62.5% had a cholestatic, 20.0% a mixed, and 17.5% a hepatocellular pattern of liver injury (Fig. 2). The median R factor in this subset was 1.4, and the mean ± SD was 7.4 ± 21.3. Seven patients had an R factor > 7, ranging from 9.19–127.

Outcomes, clinical features, and laboratory parameters for the 49 jaundiced patients overlapped substantially between those with a cholestatic, mixed, or hepatocellular injury or isolated direct hyperbilirubinemia (Table 3). However, jaundiced patients who suffered hepatocellular injury were admitted to the ICU at a higher rate than patients with cholestatic liver injury (100 *vs* 56.0%; *P* = 0.03). Patients with isolated jaundice had a higher rate of thrombocytopenia than those with cholestatic injury (100 *vs* 60%, *P* = 0.02).

**Table 3:** Outcomes and laboratory parameters in jaundiced patients with COVID‐19 and different types of liver injury

	Cholestatic	Mixed	Hepatocellular	Isolated Hyperbilirubinemia
No. of patients	25	8	7	9
ICU admission	14 (56.0%)*	7 (87.5%)	7 (100%)*	6 (66.7%)
Death	8 (32.0%)	3 (37.5%)	4 (57.1%)	5 (55.5%)
Fever	19 (76.0%)	7 (87.5%)	6 (85.7%)	7 (77.8%)
Leukopenia	6 (24.0%)	1 (12.5%)	2 (28.6%)	0 (0%)
Leukocytosis	23 (92.0%)	8 (100%)	7 (100%)	9 (100%)
Thrombocytopenia	15 (60.0%)*	5 (62.5%)	7 (100%)	9 (100%)*
Hypotension	14 (56.0%)	6 (75.0%)	5 (71.4%)	8 (88.9%)
Hypoxia	20 (80.0%)	8 (100%)	7 (100%)	8 (88.9%)
Elevated creatinine	16 (64.0%)	6 (75.0%)	5 (71.4%)	7 (77.8%)
Elevated C‐reactive protein	23 (92.0%)	8 (100%)	7 (100%)	9 (100%)
Prolonged ESR	22 (88.0%)	7 (87.5%)	6 (85.7%)	9 (100%)
Elevated procalcitonin	21 (84.0%)	6 (75.0%)	7 (100%)	8 (88.9%)
Positive blood culture	3 (12.0%)	2 (25.0%)	3 (42.9%)	4 (44.4%)
SIRS	22 (88.0%)	7 (87.5%)	7 (100%)	9 (100%)
Sepsis	3 (12.0%)	2 (25.0%)	3 (42.9%)	4 (44.4%)

* *P* < 0.05.

ESR, erythrocyte sedimentation rate; ICU, intensive care unit.

## Discussion

Since SARS‐CoV‐2 was first identified, there has been a rapidly emerging body of literature describing the nature of COVID‐19. Many studies have explored liver injury and liver blood test abnormalities; however, hepatic dysfunction manifested by jaundice in patients with COVID‐19 has not been a primary focus. Unlike liver injury as manifested by elevated serum aminotransferase or ALP activities, elevated serum direct bilirubin concentrations and jaundice are indicators of hepatic secretory dysfunction. Liver function may be completely normal in an individual with significantly elevated serum ALT and ALP activities; conversely, liver function may be abnormal in individuals with normal or near‐normal serum activities of these enzymes. We therefore described the outcomes and features of patients hospitalized with COVID‐19 with liver dysfunction as manifested by jaundice. We found that 8.9% of the hospitalized patients with COVID‐19 in our cohort had hepatic jaundice, consistent with previous studies reporting jaundice in 6.0–16.7% patients.^12^ Our analysis then specifically looked at mortality associated with hepatic jaundice and found it to be 40.8%, a case fatality rate similar to what has been reported for patients with COVID‐19 requiring invasive mechanical ventilation.^13^, ^14^ We also found that the ICU admission rate was approximately 2.5 times higher for patients with hepatic jaundice. Hence, jaundice is an ominous sign in patients with COVID‐19.

Most studies of liver injury in patients with COVID‐19 primarily focused on serum aminotransferase and ALP activities. Some considered serum bilirubin concentrations in their analysis of patient outcomes, although not elevated to the degree or appropriately fractionated to be consistently indicative of hepatic jaundice. One meta‐analysis of several such studies revealed conflicting results regarding correlations between disease severity and TBIL.^15^ In our initial investigation of liver injury in patients hospitalized with COVID‐19, we found that a TBIL or serum direct bilirubin concentration above the ULN on admission or subsequently during hospitalization was associated with increased mortality.^7^ In one retrospective cohort study of 1788 patients hospitalized with COVID‐19 in Wuhan, China, mortality was considerably higher among those with TBIL above the ULN.^16^ Additionally, serum procalcitonin and creatinine concentrations and platelet counts differed significantly between those with and without elevated TBIL, similar to what we found in our analysis. Another study from Wuhan during the first few months of the pandemic also found that mortality was associated with TBIL above the ULN both on admission and at peak value during hospitalization, even though elevations were relatively uncommon within the entire cohort.^17^ Additionally, the authors noted that a direct bilirubin concentration above the ULN was commonly found during the hospital stay of patients who died. In a study of patients with COVID‐19 from a hospital system in the northeastern United States, an abnormally elevated TBIL during hospitalization was associated with increased odds of ICU admission, mechanical ventilation, and death.^18^ In a cohort of patients admitted with COVID‐19 to a tertiary care hospital in the Bronx, New York, mean TBIL trended higher in patients who died than in those who survived; however, the difference did not reach statistical significance.^19^ In another study of only 65 patients with COVID‐19 from an academic hospital in Houston, Texas, there was similarly a nonsignificant trend toward a greater frequency of TBIL elevations in those who died.^20^

SARS‐CoV‐2 infection in and of itself does not appear to be a cause of jaundice. Even in our entire cohort of hospitalized patients with approximately 90% meeting criteria for SIRS, fewer than 9% were jaundiced. Our data show that jaundice in patients with COVID‐19 is associated with fever, leukopenia, leukocytosis, thrombocytopenia, hypotension, and hypoxemia. Jaundiced patients had an increased frequency of elevated serum creatinine concentration, indicative of acute kidney injury, as well as higher rates of elevated serum procalcitonin and positive blood cultures. These findings strongly suggest that complicating bacterial sepsis or severe systemic inflammation, beyond what meets the criteria for SIRS, are the major causes of jaundice in patients with COVID‐19.

Sepsis is a common cause of jaundice, especially cholestatic jaundice or isolated hyperbilirubinemia.^21^, ^22^, ^23^, ^24^ Of all the jaundiced patients with COVID‐19 in this cohort, 51.0% had a cholestatic pattern of liver injury, and 18.4% had isolated hyperbilirubinemia. We considered a patient to be septic if they met the criteria for SIRS and had positive blood cultures. Based on these criteria, the percentage of any jaundiced patient with COVID‐19 having sepsis was 24.5%, triple that of non‐jaundiced patients. Blood cultures are the gold standard for detection of bacteremia; however, they may miss it, especially in patients already receiving antibiotics.^25^ At our institution, antibiotics were used liberally during the early stages of the COVID‐19 pandemic out of concern for superimposed bacterial pneumonia, which may have impacted the rate of positive blood cultures. In recent years, serum procalcitonin concentration has emerged as a potential marker of bacterial infection.^26^, ^27^ In our cohort, 85.7% of jaundiced patients had a serum procalcitonin concentration above the ULN at some point during hospitalization, compared with 61.6% of non‐jaundiced patients. However, increased serum concentrations of procalcitonin can be attributable to noninfectious insults, including potentially hepatic dysfunction.^28^ Nonetheless, sepsis appears to be significantly more common among COVID‐19 patients with jaundice than those without.

Another etiology of conjugated hyperbilirubinemia and jaundice is severe hepatocellular injury, including that which occurs in ischemic/hypoxic hepatitis.^29^ In our previous study of this entire patient cohort, we identified 21 patients who suffered severe hepatocellular injury, which commonly led to elevated TBIL.^7^ In some of these patients, we identified hypotension along with evidence of acute renal failure. Of the jaundiced patients in the current study, seven (14.3%) had evidence of hepatocellular injury, which was likely severe as all of them had a very high R factor and six had an ALT >1000 U/L. Each of these patients was admitted to the ICU and had evidence of SIRS; 42.9% met the criteria for sepsis, 100% suffered from hypoxia, and 71.4% were hypotensive. Hence, ischemic/hypoxic hepatitis is another likely cause of jaundice in hospitalized patients with COVID‐19.

This study had several limitations. We used a retrospective observational cohort design with inclusion restricted to patients hospitalized at a single medical center with an encounter diagnosis of COVID‐19. This may have led to a selection bias according to how providers documented diagnoses in the electronic medical record. The association of jaundice with sepsis is challenging because the pathobiology of sepsis is still uncertain, and it can only be identified by a constellation of clinical signs and symptoms in a patient with suspected infection. The use of SIRS criteria to define sepsis has been challenged, as many hospitalized patients meet these criteria, including those who never develop infection.^30^ Basing a diagnosis of sepsis on SIRS criteria may also miss cases.^31^ Certain factors such as preexisting comorbidities, simultaneous illnesses, and medications could also have contributed to clinical features, laboratory test results, and outcomes that we were unable to account for in our analysis. Finally, we characterized hepatic injury pattern using the R factor, a metric originally developed for drug‐induced liver injury. However, an American College of Gastroenterology Clinical Guideline has recommended that the R factor be used more broadly to characterize liver injury.^32^

In conclusion, hospitalized patients with COVID‐19 who have hepatic jaundice have significantly more adverse outcomes, including increased rates of ICU admission and death. Hepatic jaundice in patients with COVID‐19 has a mortality rate similar to that observed in patients receiving invasive mechanical ventilation. The main etiologies of liver dysfunction leading to conjugated hyperbilirubinemia and jaundice appear to be sepsis, severe systemic inflammation greater than that of typical SIRS, and hypoxic/ischemic hepatitis.
